# Supplementary material for: Stabilizing lattice oxygen redox in layered sodium transition metal oxide through spin singlet state
Source: Nat Commun. 2023 Nov 23;14:7665. doi: 10.1038/s41467-023-43031-6 (PMC10667238; doi:10.1038/s41467-023-43031-6)
Supplement: Supplementary file 1 — Supplementary Information [file 41467_2023_43031_MOESM1_ESM.pdf]

Supporting information

Stabilizing Lattice Oxygen Redox in Layered Sodium Transition Metal Oxide Through Spin Singlet State

Xuelong Wang[1,2], Liang Yin[2,3], Arthur Ronne[1,4], Yiman Zhang[5], Zilin Hu[2], Sha Tan[1], Qinchao Wang[1], Bohang Song[6], Mengya Li[7], Xiaohui Rong[2], Saul Lapidus[3], Shize Yang[8], Enyuan Hu[1,*], Jue Liu[6,*]

[1] Chemistry Division, Brookhaven National Laboratory, Upton, NY 11973, United States

[2] Institute of Physics Chinese Academy of Sciences, 100190 Beijing, China

[3] X-ray Science Division, Advanced Photon Source, Argonne National Laboratory, Argonne, IL 60439, United States

[4] Department of Materials Science and Chemical Engineering, Stony Brook University, Stony Brook, NY 11794, USA

[5] Chemical Sciences Division, Oak Ridge National Laboratory, Oak Ridge, TN 37831, USA

[6] Neutron Scattering Division, Oak Ridge National Laboratory, Oak Ridge, TN 37922, USA

[7] Electrification and Energy Infrastructure Division, Oak Ridge National Laboratory, Oak Ridge, TN 37830, USA

[8] Energy Sciences Institute, Yale University, 810 West Campus Drive, West Haven, CT,06516, United States

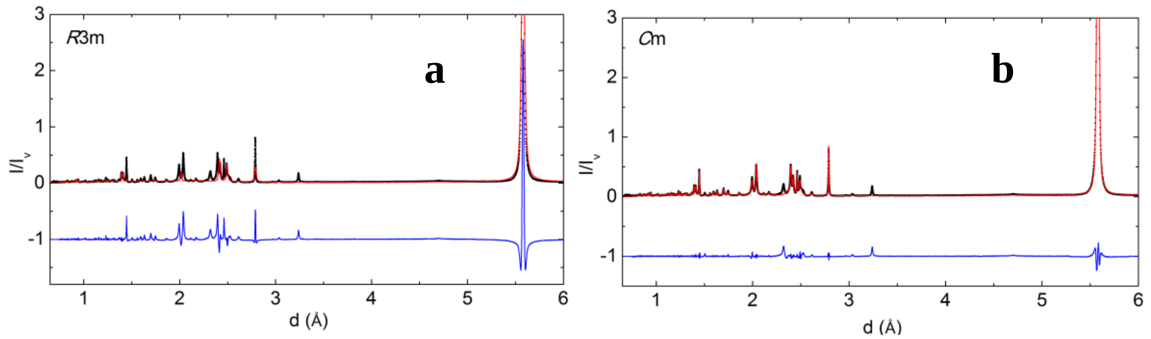

Supplementary Fig. 1 Le Bail fits of the P3-type $Na_{2/3}Cu_{1/3}Mn_{2/3}O_2$ using high resolution XRD (λ = 0.41285 Å) with (a) S.G. *R*3m (R_{wp} =44.4%, Gof =10.44) and (b) S.G. *C*m (R_{wp} =16.9% , Gof = 4.00).

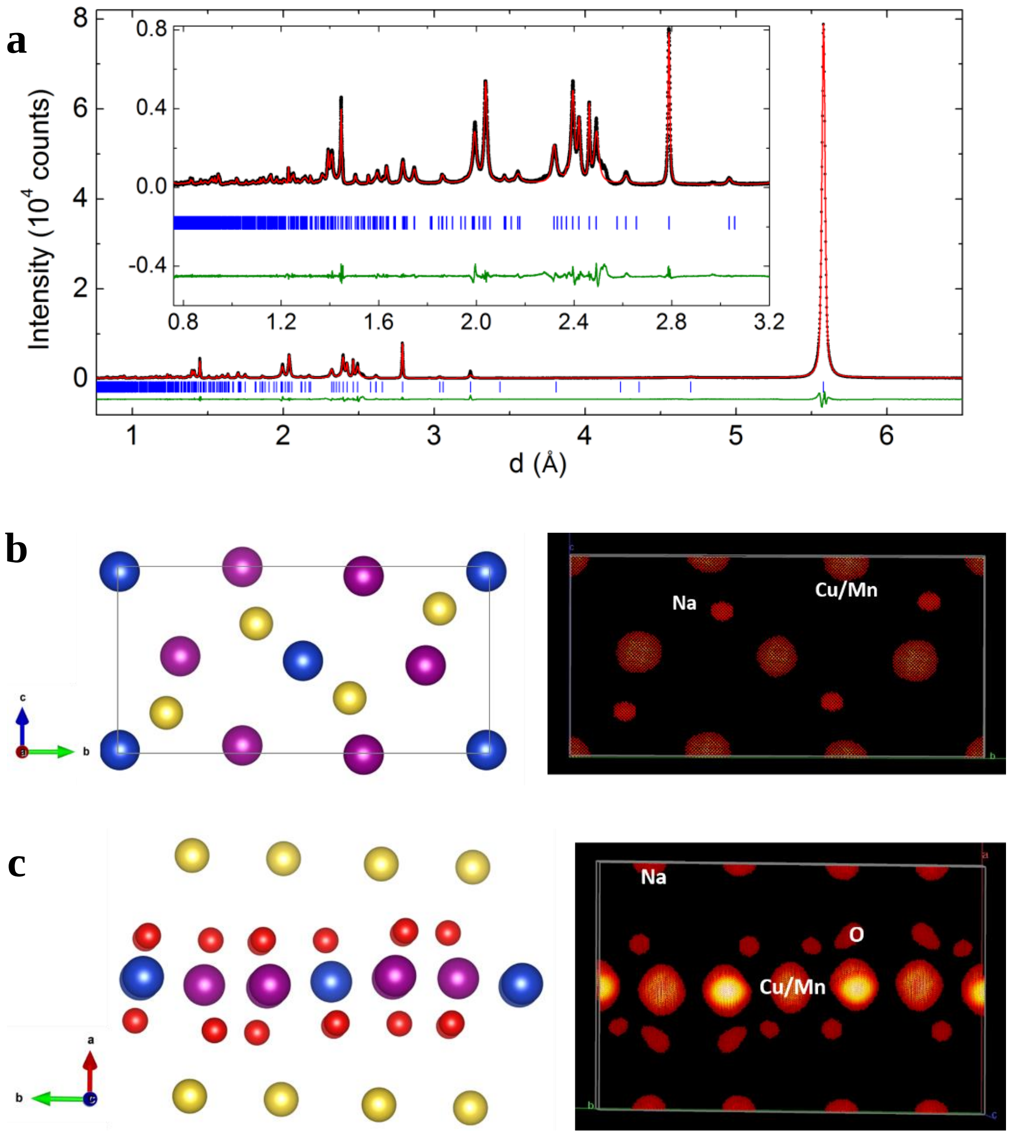

Supplementary Fig. 2 (a) Pattern indexing, and Le Bail fit of the P3-type $Na_{2/3}Cu_{1/3}Mn_{2/3}O_2$ using synchrotron XRD (S.G. $P2_1/c$, R_{wp} =9.53%, Gof =2.27). Note the extra peak around 2.4 Å is from the CuO impurity. (b)-(c) Charge-flipping results showing the electron densities (right) of plausible atomic positions, compared to those refined from Rietveld refinement on the left (Na, O, Cu and Mn are shown in yellow, red, blue and purple balls). Higher threshold (without showing oxygen electron density) is used for the electron density map in the *bc*-plane (b) for clarity. Cu and Mn is indistinguishable because of their very similar atomic scattering factors. While positions for metals (Na, Cu and Mn) identified by charge flipping are very close to those in the final Rietveld refinement, the initial electron density maximum positions of oxygen ions are quite off from the refined positions (c), presumably due to the low electron density (atomic scattering factor) of oxygen.

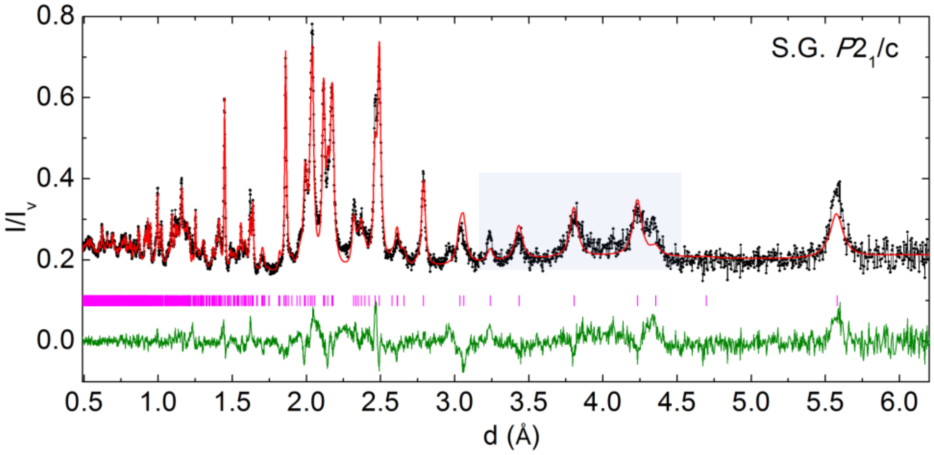

Supplementary Fig. 3 Structure refinement of the P3-type $Na_{2/3}Cu_{1/3}Mn_{2/3}O_2$ (S.G. $P2_1/c$) using high resolution neutron diffraction data from POWGEN. It is worth noting there is obvious intensity mismatches in the Bragg reflections above 3.0 Å. This is due to the stacking disorder of honeycomb ordered Cu-Mn layers along the stacking direction. A numerical stacking disorder model that can reasonably describe this phenomenon is shown in Figure S5.

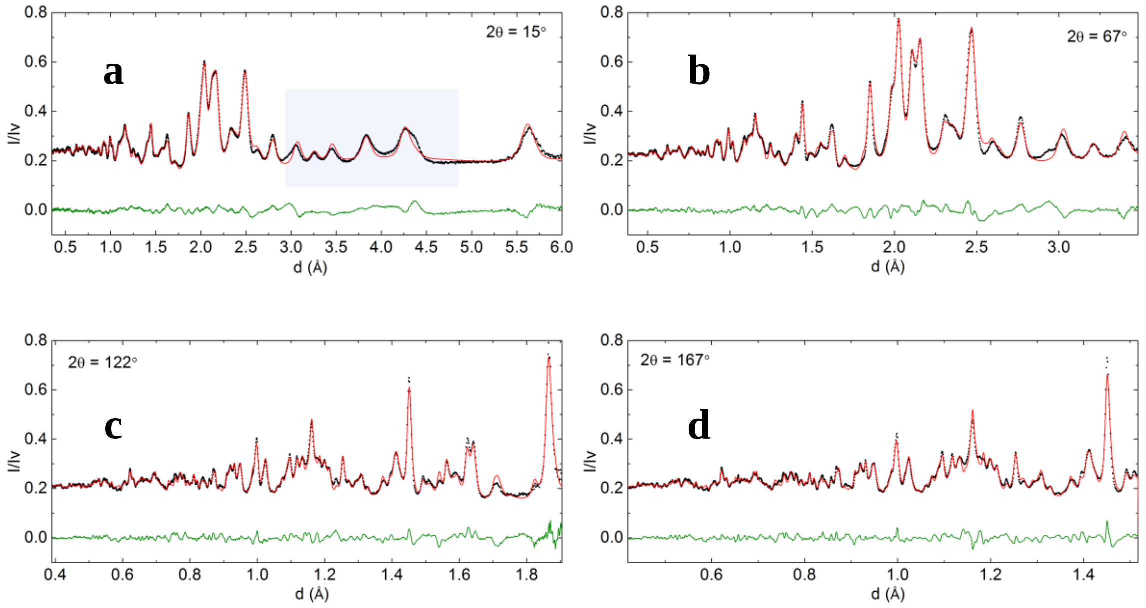

Supplementary Fig. 4 (a-d) Structure refinement results of the P3-type $Na_{2/3}Cu_{1/3}Mn_{2/3}O_2$ using neutron diffraction data from NOMAD at different banks. Similar discrepancies have been observed as those in the POWGEN data.

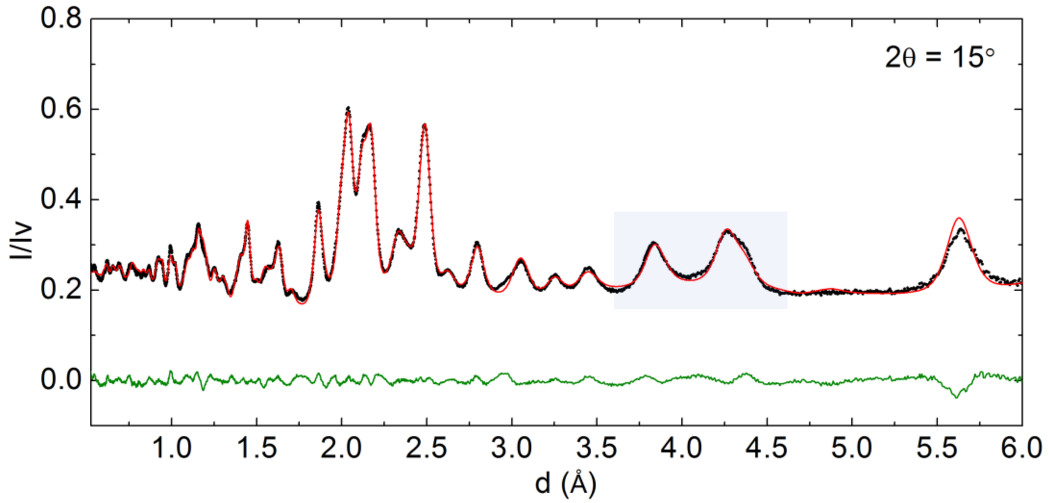

Supplementary Fig. 5 Structure refinements of P3-type $Na_{2/3}Cu_{1/3}Mn_{2/3}O_2$ using the numerical stacking disorder model of the honeycomb ordered Cu-Mn layers (note, the stacking direction is along *a*-axis in the current $P2_1/c$ configuration). Only the low-angle bank is used here because the most obvious discrepancies are from the high d-spacing super lattice peaks (highlighted in the figure). The structure that provides the best fit is found to have ~15% stacking disorder, i.e. 85% (neighboring) layers with the translation vectors (x,y) of (0,0) and 15% with the translation vector of (0,1/3). To generate the stacking sequences, a total sequence number of 100 is used and the number of stacks per sequence is also set to be 100. The atomic coordination (x and y) is fixed to the values obtained from Rietveld refinement, the z coordination is set to the values of z_0/N_v, where z_0 is the z coordinates obtained from Rietveld refinement (which is the x coordination in the S.G. $P2_1/c$ configuration or the z coordination in the S.G. $P2_1/a$ configuration). Only lattice parameters and isotropic atomic displacements are allowed to refine. Noticeable improvements can be seen, especially for the super lattice peaks associated with Cu-Mn honeycomb ordering. The final R_{wp} is 2.6% and Gof is 3.02.

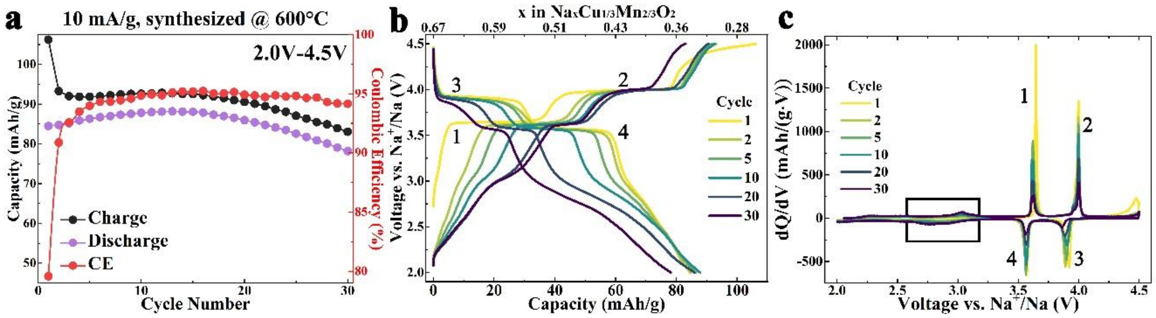

Supplementary Fig. 6 (a) Charge, discharge capacity and coulombic efficiency of the 600℃-synthesized P3-type $Na_{2/3}Cu_{1/3}Mn_{2/3}O_2$ sample between 2.0V and 4.5V (versus Na^+/Na) for 30 cycles. (b) The charge and discharge curves of cycle 1, 2, 5, 10, 20 and 30 between 2.0 V and 4.5 V (versus Na^+/Na). The voltage profile quickly degrades as cycling prolongs with capacity shrinking, voltage fading and low voltage plateau emerging which may indicate structural failure and increasing capacity contribution from the Mn redox; (c) the differential voltage (dQ/dV) curves of corresponding cycles where peak intensity quickly decreases with prolonged cycles and new peaks at low voltage begin to rise (circled out by black line), all of which indicate a systematic material degradation.

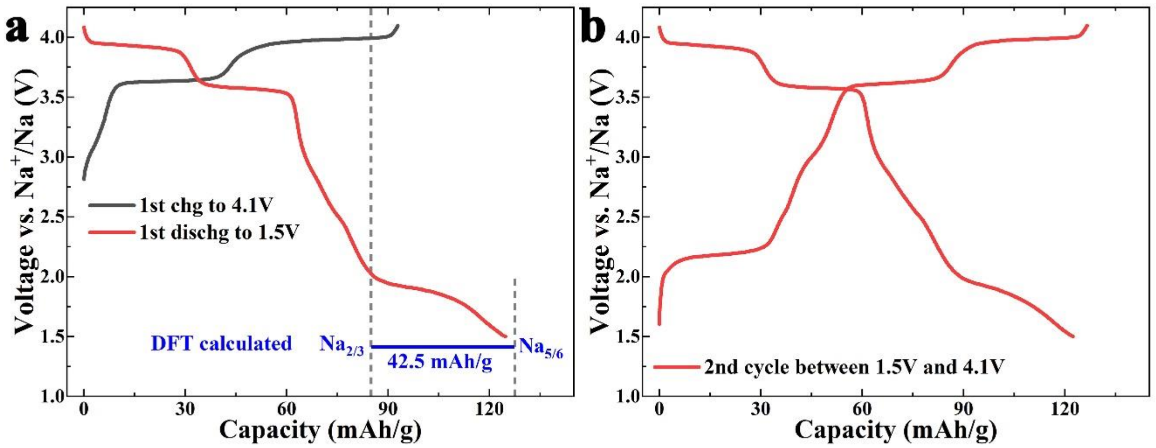

Supplementary Fig. 7 (a) The voltage curve of 1st charging to 4.1 V and 1st discharging to 1.5 V with blue line marking the average voltage predicted by DFT calculation for the excessive sodiation of P3-type $Na_{2/3}Cu_{1/3}Mn_{2/3}O_2$ to $Na_{5/6}Cu_{1/3}Mn_{2/3}O_2$ activating Mn redox. Qualitative consistency is reached between DFT prediction and electrochemical measurement in that voltage for Mn redox is lower than 2.0 V, different from other P-type sodium ion cathode with active Mn redox between 2.0 V and 3.0 V. The quantitative discrepancy between measured voltage and DFT prediction is a result of insufficient Na-vacancy configuration sampling in DFT calculation. (b) The voltage curve of 2nd charge-discharge cycle between 1.5 V and 4.1 V, which shows good reversibility of capacity and voltage plateaus. The low voltage plateau corresponding to Mn redox shows larger hysteresis between charge and discharge than those above 3.5 V suggesting poor kinetic of the sodiation process in activating Mn redox.

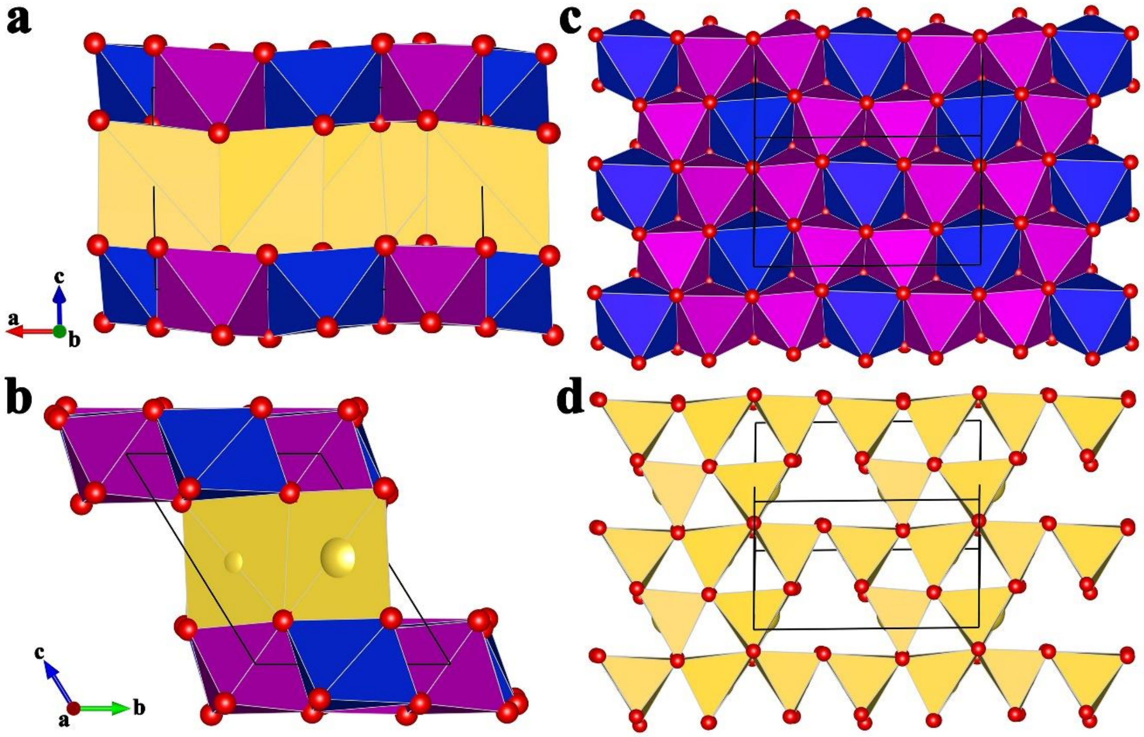

Supplementary Fig. 8 The illustrative showing of structural model used by DFT calculation for excessively sodiated state $Na_{5/6}Cu_{1/3}Mn_{2/3}O_2$ in (a-b) side view, top view of (c) Cu-Mn layer and top view of (d) Na layer with Na^+ ions occupying the prismatic sites and sharing faces with TM cations.

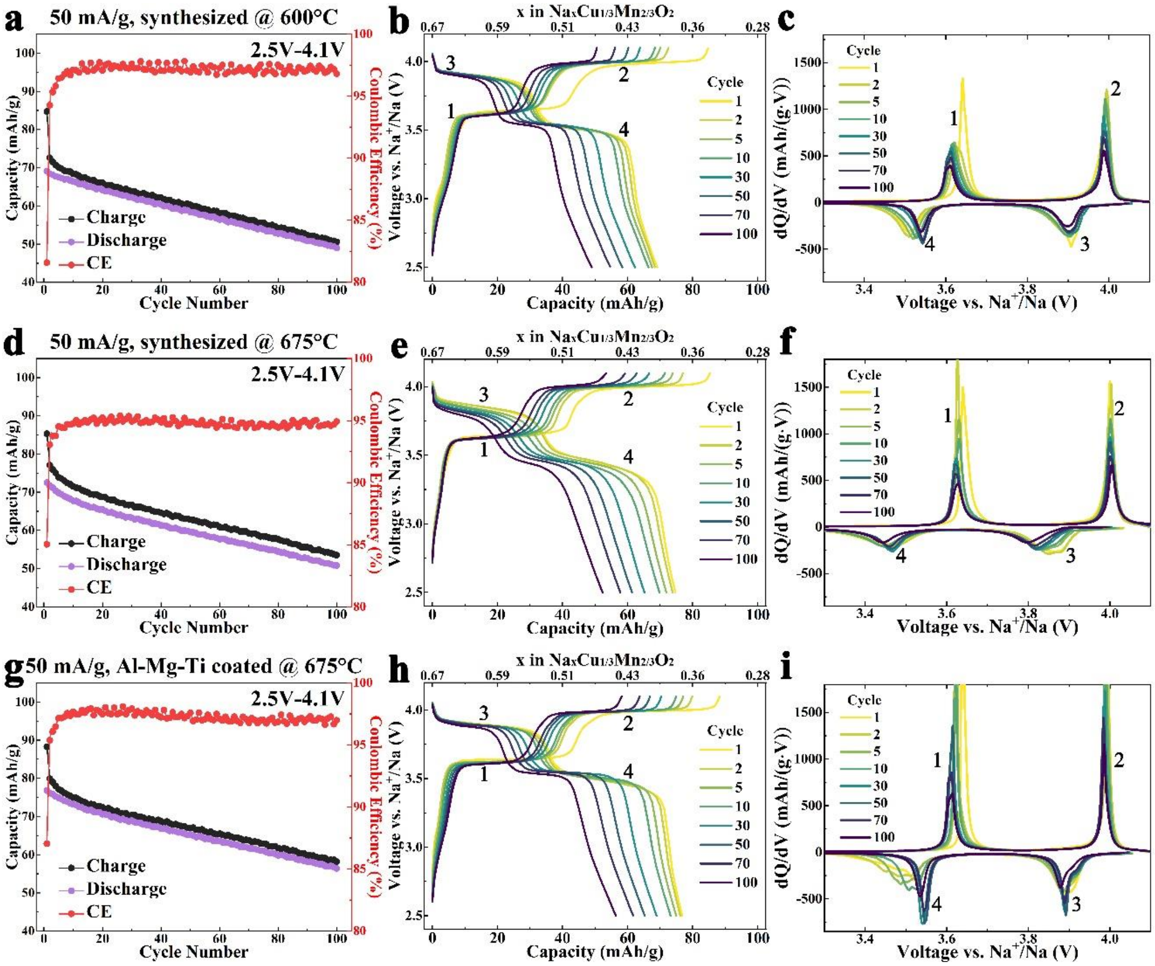

Supplementary Fig. 9 The 50 mA/g cycling performances, representative voltage profiles, and corresponding differential voltage curves of P3-type $Na_{2/3}Cu_{1/3}Mn_{2/3}O_2$ samples synthesized at different conditions: (a-c) a regular 600℃ solid-state synthesis; (d-f) solid-state synthesis at elevated temperature of 675℃; (g-i) solid-state synthesis at 675℃ with multi-element trace doping of Al, Mg, and Ti.

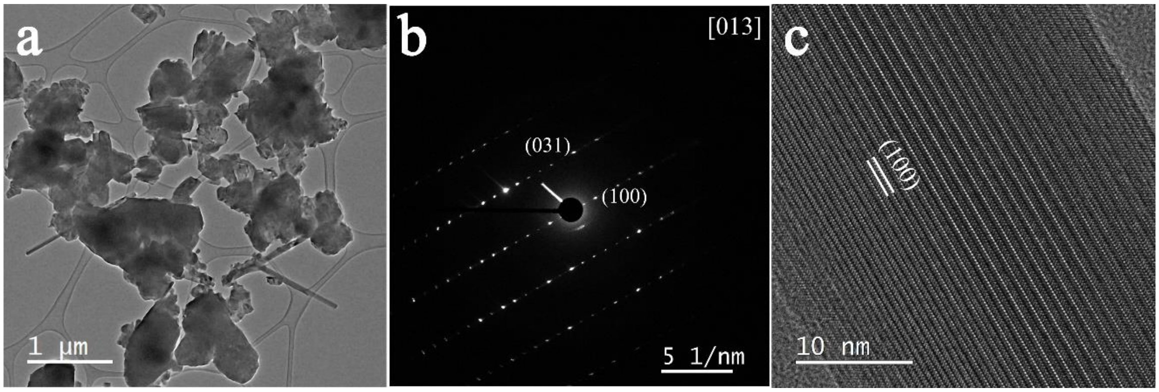

Supplementary Fig. 10 (a) Low magnification TEM images showing the morphology of the synthesized P3-type $Na_{2/3}Cu_{1/3}Mn_{2/3}O_2$ material particles. (b) Electron diffraction pattern of selected area along the [013] direction with the marked spots corresponding to the (100) and (031) diffraction plane. (c) High resolution TEM image showing the cross section of (100) lattice planes in the P3-type $Na_{2/3}Cu_{1/3}Mn_{2/3}O_2$ particle which demonstrates the perfect layered structure of synthesized samples.

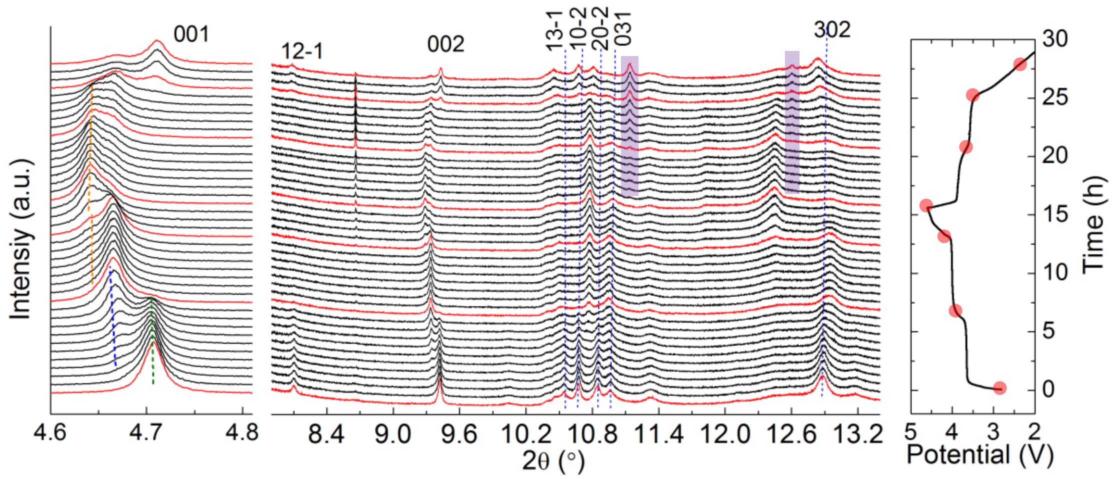

Supplementary Fig. 11 *In situ* XRD of P3-type $Na_{2/3}Cu_{1/3}Mn_{2/3}O_2$ versus Na^+/Na with the upcut-off voltage of 4.5 V (versus Na^+/Na). The corresponding charge/discharge data is shown on the right. Critical phase transition points are highlighted in red colored curves and key Bragg reflections are labeled on the top of the diffraction patterns.

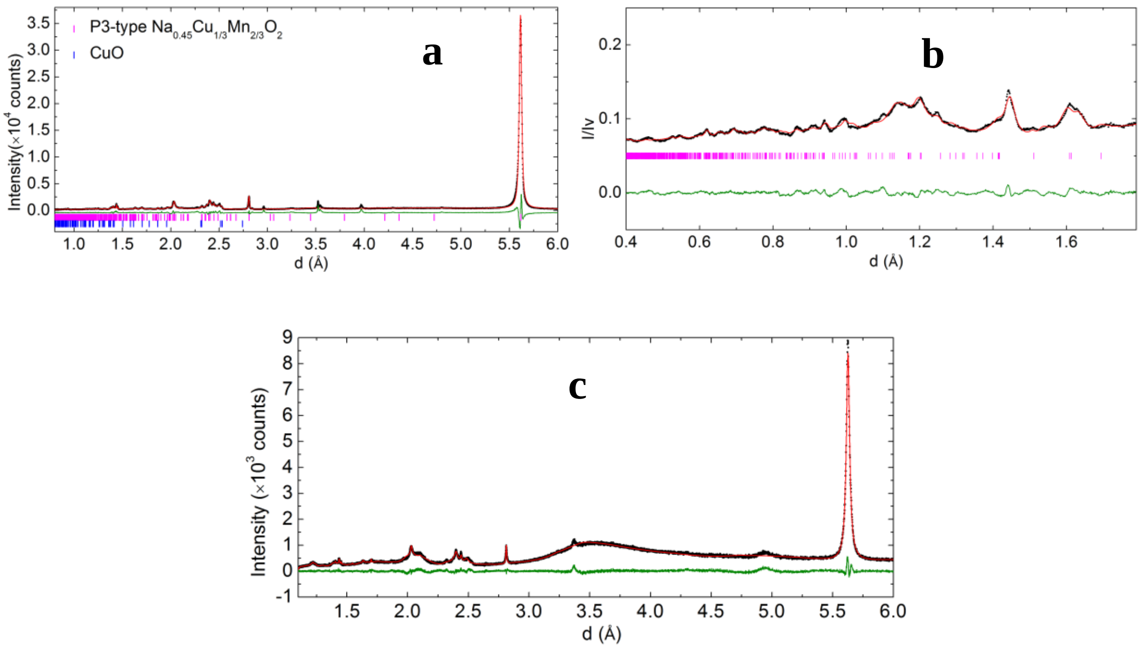

Supplementary Fig. 12 Rietveld refinement of the structure of 3.66 V charged $Na_xCu_{1/3}Mn_{2/3}O_2$ using (a) *ex situ* synchrotron XRD data, (b) *ex situ* neutron diffraction data (bank 5, center 2θ = 167°) and (c) *in situ* synchrotron XRD data.

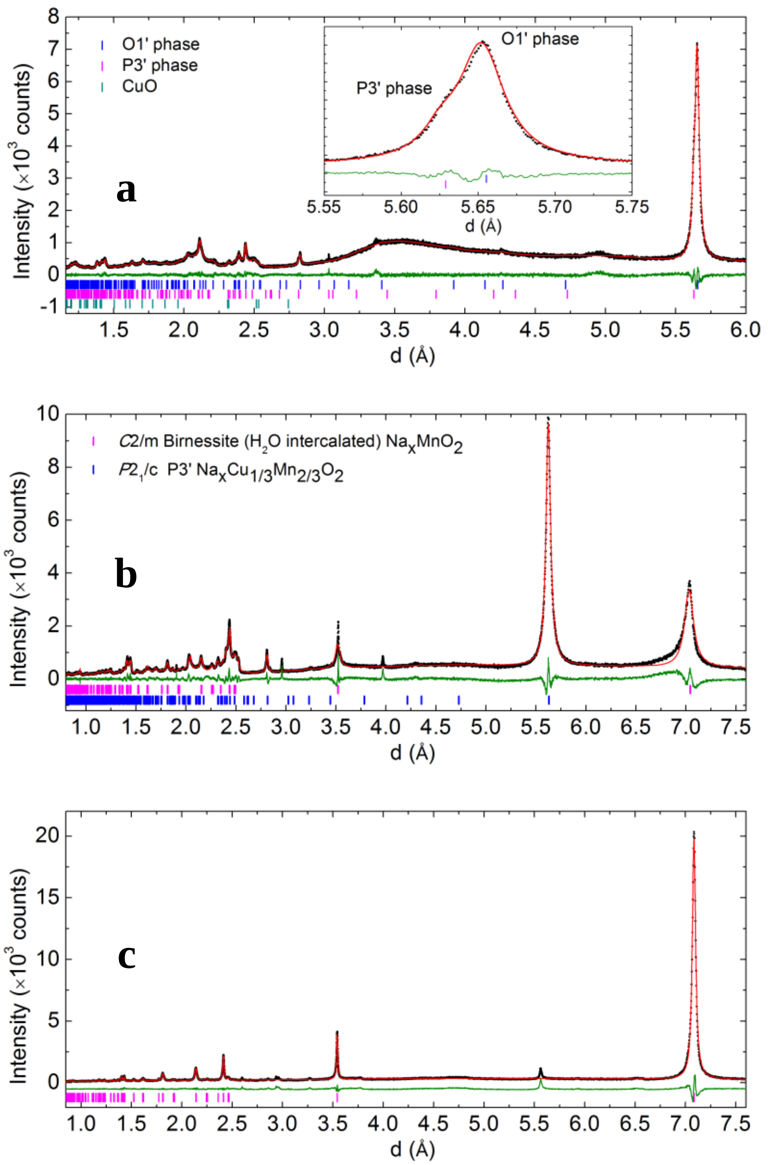

Supplementary Fig. 13 (a) Rietveld refinement of the structure of the 4.6 V (versus Na^+/Na) charged $Na_xCu_{1/3}Mn_{2/3}O_2$ using *in situ* synchrotron XRD data. Two phases co-exist (P3' and O1' phases) when charged to 4.6 V. During the data collection of the fully charged ex situ sample, we found that the O1' phase can easily transform to another phase with a very large d-spacing peak located at about 7 Å (b) (sealed in the Kapton capillary and only exposed to the air during data collection). This is likely due to the structure relaxation and the absorption of moisture, similar phenomenon was observed for the desodiadiated $Na_{2-x}Mn_3O_7$ (c). The water intercalated phase becomes more crystalline, as can be seen from the much sharper Bragg peak. This highlights the importance of monitoring the structure changes using *in situ* diffraction.

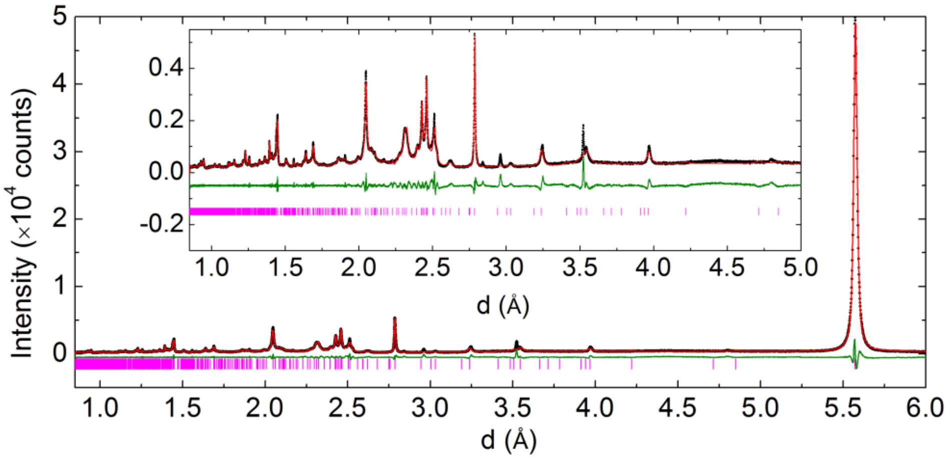

Supplementary Fig. 14 Le Bail fit of the structure of the fully discharged $Na_xCu_{1/3}Mn_{2/3}O_2$ (2.0 V versus Na^+/Na) using *ex situ* synchrotron XRD data.

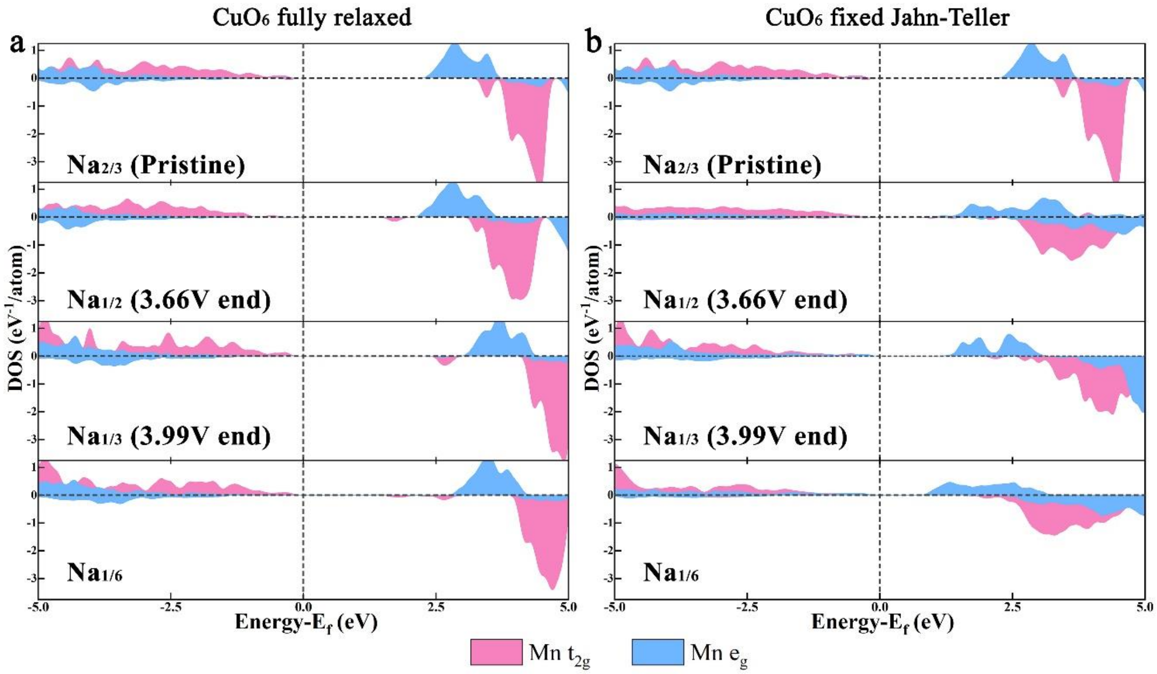

Supplementary Fig. 15 DFT calculated pDOS of Mn d orbitals of P3- type $Na_{2/3}Cu_{1/3}Mn_{2/3}O_2$ at different desodiation states for models with (a) fully relaxed and (b) Jahn-Teller distorted CuO_6 octahedron.

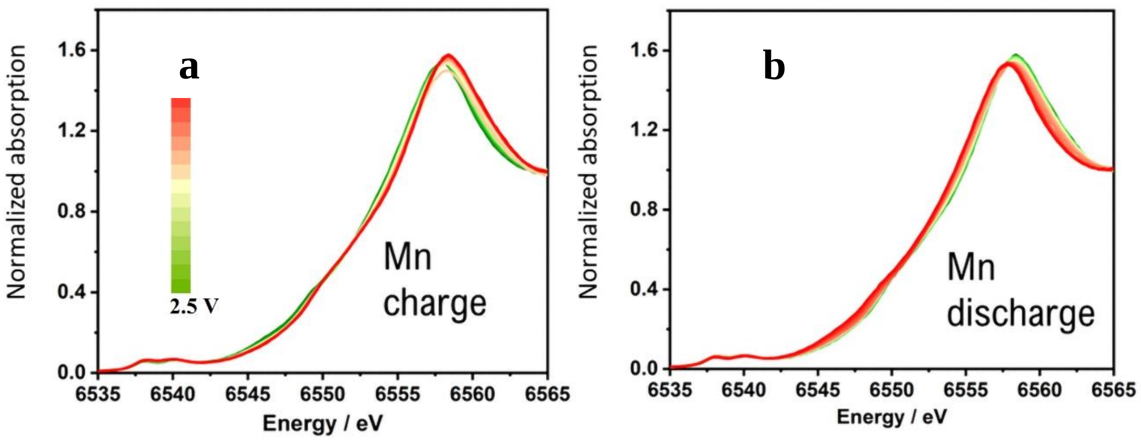

Supplementary Fig. 16 In-situ XANES spectra at Mn K-edge of P3-type $Na_{2/3}Cu_{1/3}Mn_{2/3}O_2$ during (a) charging and (b) discharging of 1st cycle.

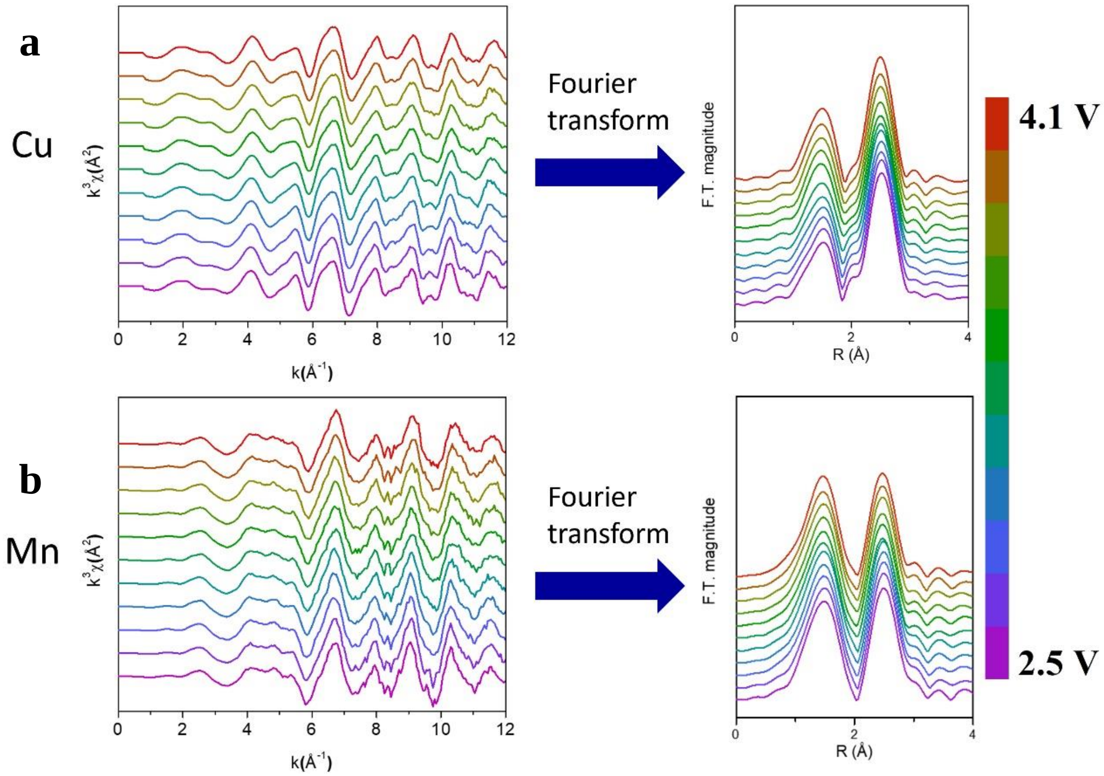

Supplementary Fig. 17 Fourier transformed spectra in reciprocal and real space of in-situ EXAFS data at (a) Cu and (b) Mn K-edge during charging for P3-type $Na_{2/3}Cu_{1/3}Mn_{2/3}O_2$.

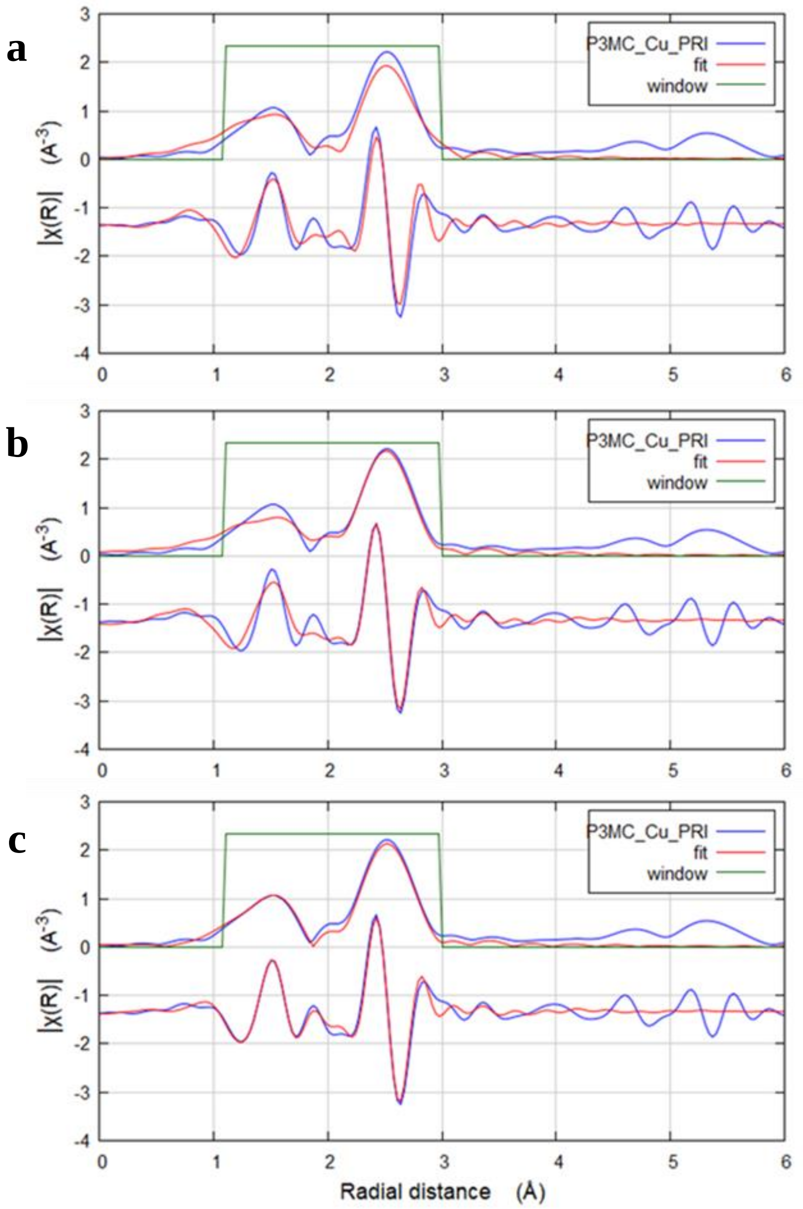

Supplementary Fig. 18 Results of fitting the magnitude of the Fourier transformed EXAFS (weighted by k^3). (a) No local structural feature is considered; (b) Only honeycomb ordering between Cu and Mn is considered; (c) both honeycomb ordering and Jahn-Teller distortion of CuO_6 octahedron are considered.

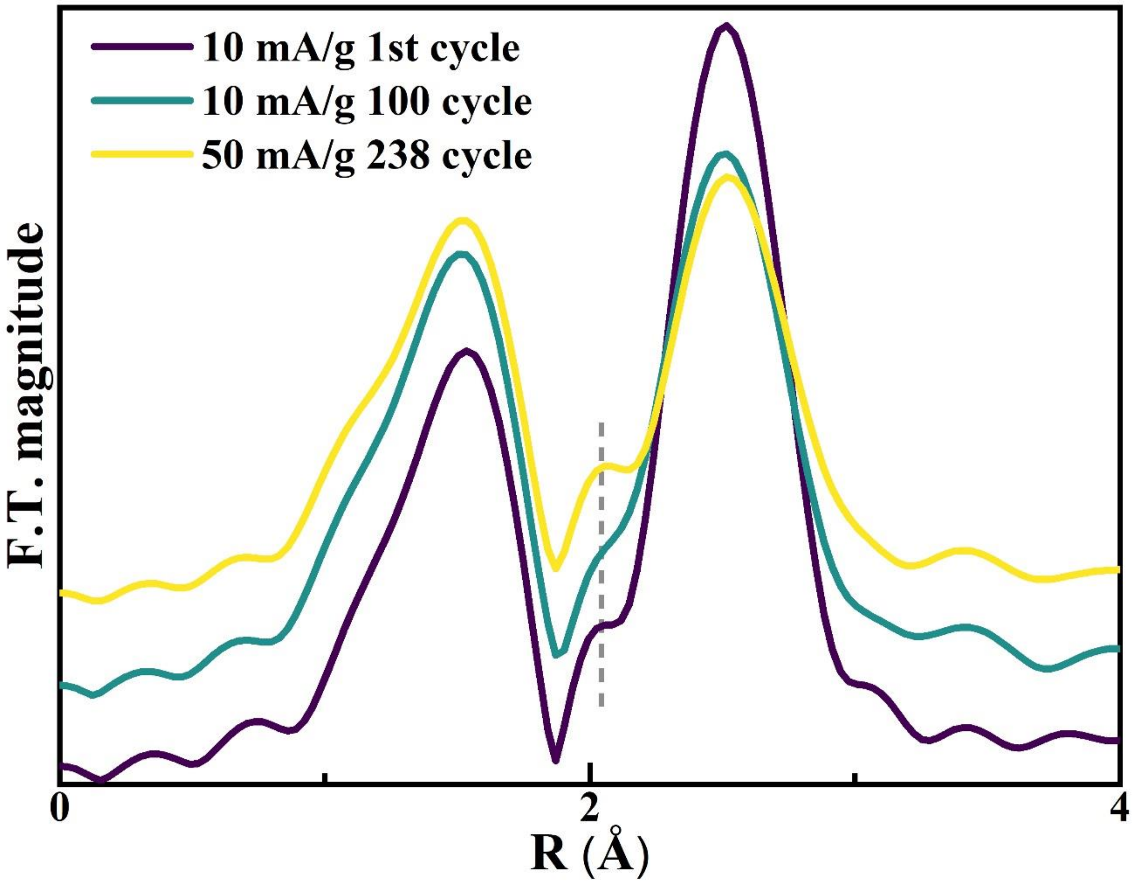

Supplementary Fig. 19 Comparison between Fourier transformed EXAFS spectra in real space at Cu K-edge of samples after different cycles. Dotted grey line marks the side peak corresponding to the short Cu-O bond in JT-distorted CuO_6 octahedron which suggests the preservation of JT distortion in P3-type $Na_{2/3}Cu_{1/3}Mn_{2/3}O_2$ after long-term cycling.

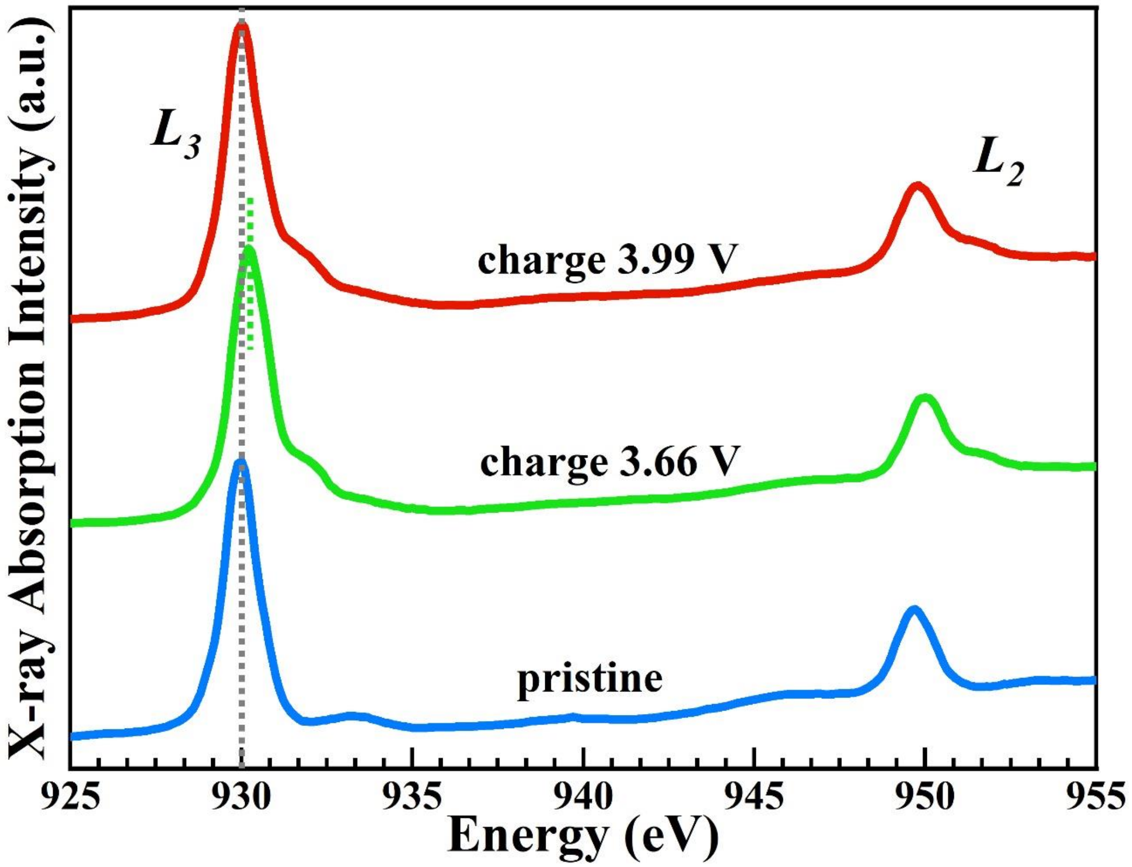

Supplementary Fig. 20 The full energy range Ex-situ XANES spectra at Cu L-edge.

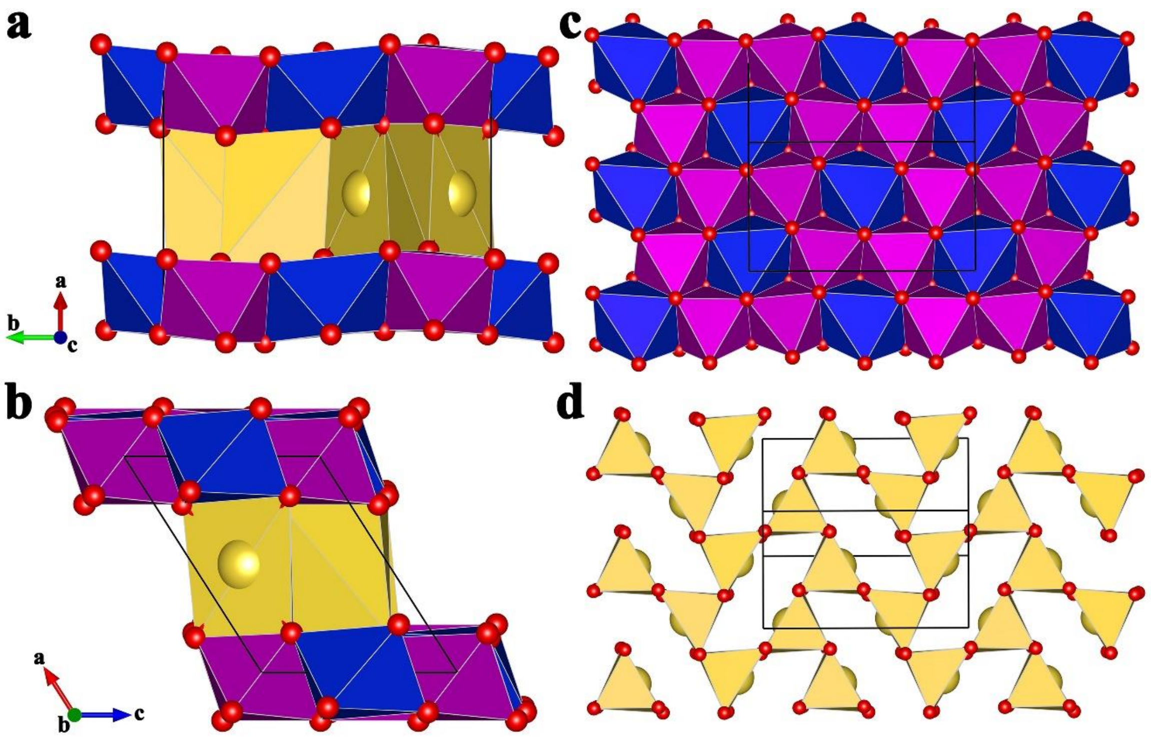

Supplementary Fig. 21 The illustrative showing of structural model used by DFT calculation for pristine P3-type $Na_{2/3}Cu_{1/3}Mn_{2/3}O_2$ in (a-b) side view, top view of (c) Cu-Mn layer and top view of (d) Na layer with Na^+ ions occupying the prismatic sites.

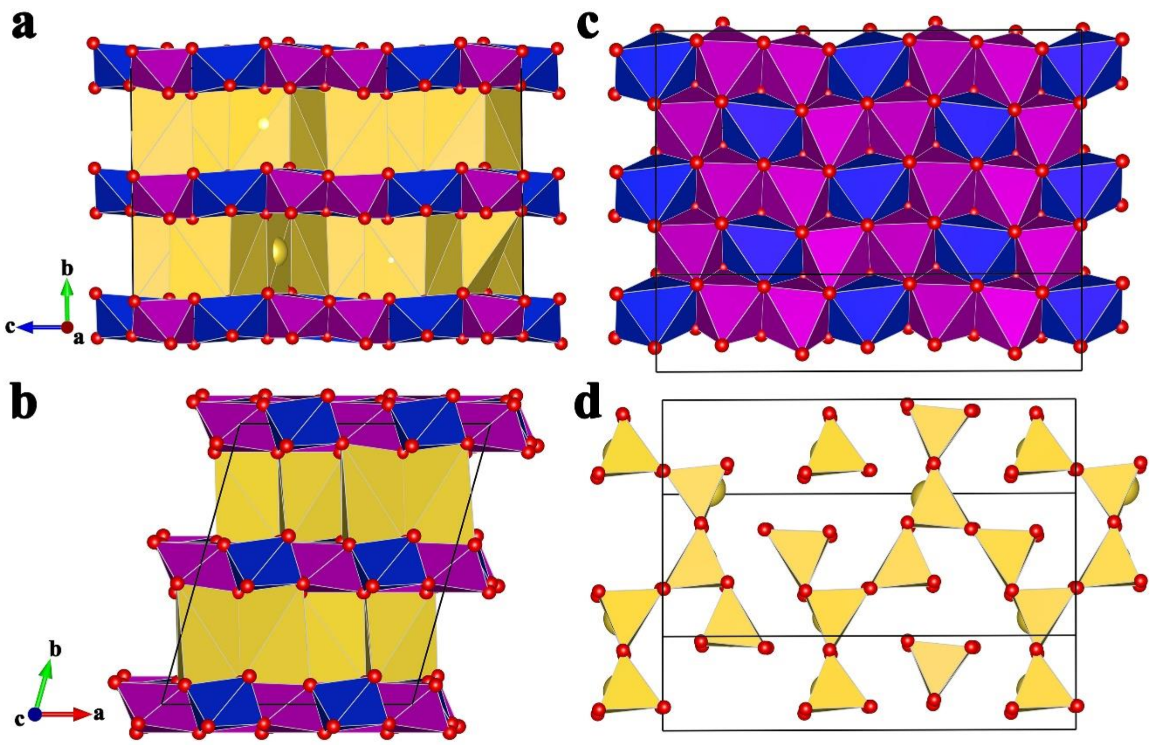

Supplementary Fig. 22 The illustrative showing of supercell model used by DFT calculation for P3' $Na_{1/2}Cu_{1/3}Mn_{2/3}O_2$ in (a-b) side view, top view of (c) Cu-Mn layer and top view of (d) Na layer with Na^+ ions occupying the prismatic sites.

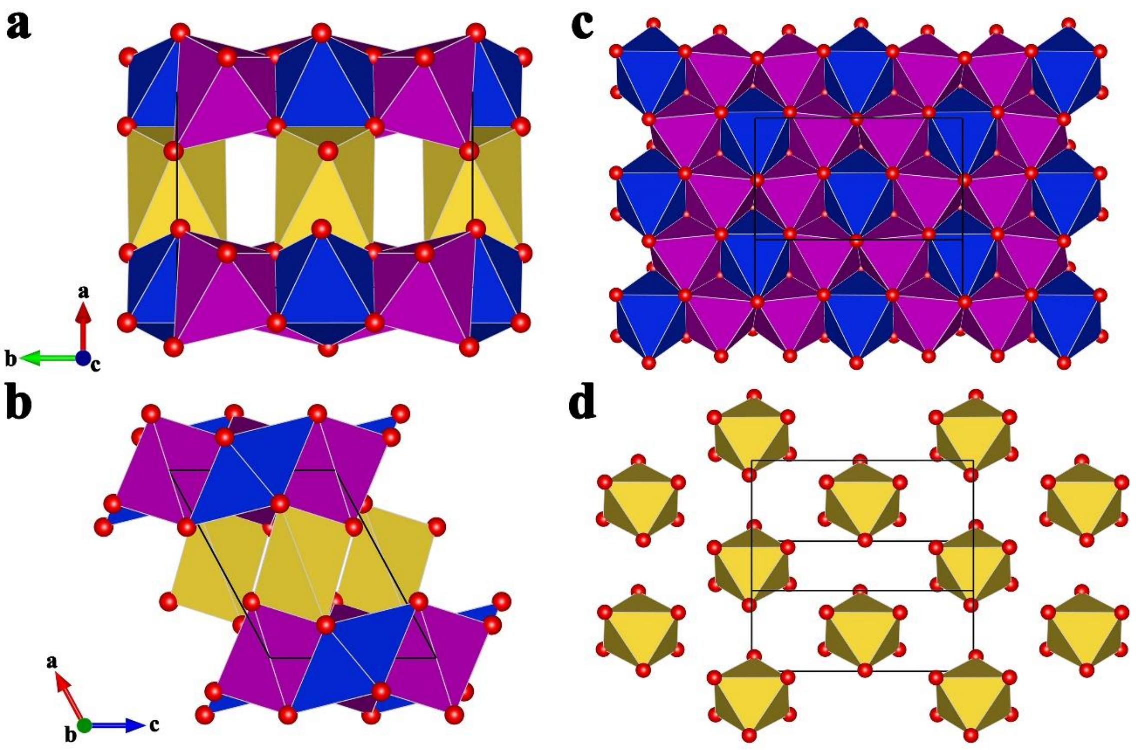

Supplementary Fig. 23 The illustrative showing of structural model used by DFT calculation for O1’ $Na_{1/3}Cu_{1/3}Mn_{2/3}O_2$ in (a-b) side view, top view of (c) Cu-Mn layer and top view of (d) Na layer with Na^+ ions occupying the octahedral sites.

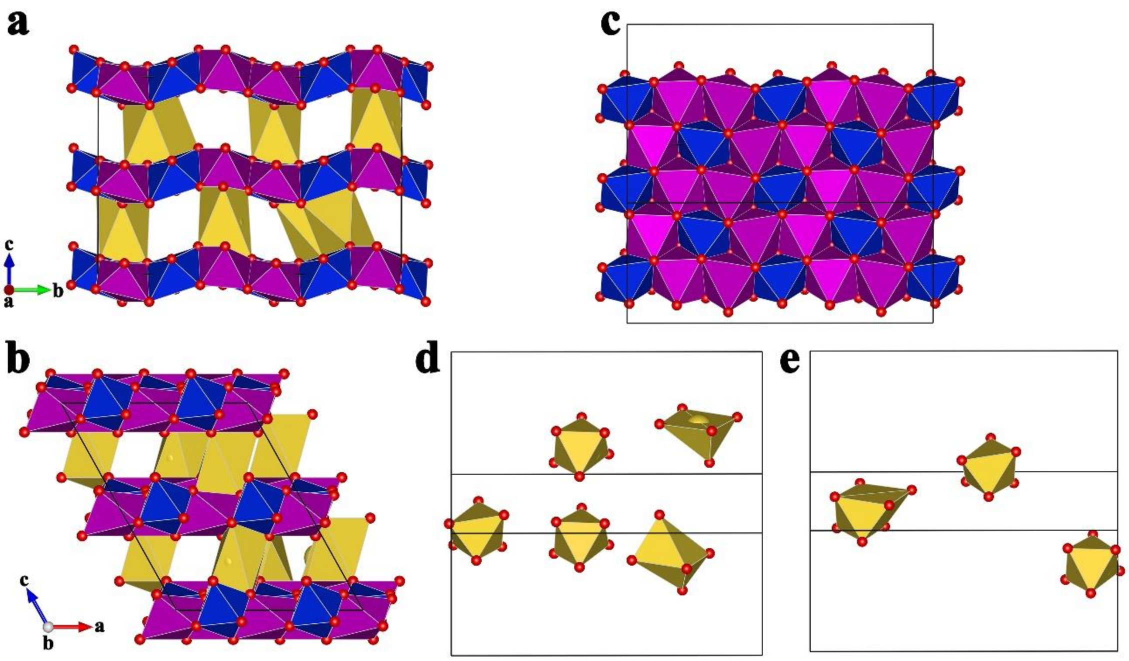

Supplementary Fig. 24 The illustrative showing of structural model used by DFT calculation for O1’ $Na_{1/6}Cu_{1/3}Mn_{2/3}O_2$ in (a-b) side view, top view of (c) Cu-Mn layer and top view of (d) 1st Na layer and € 2nd Na layer with Na^+ ions occupying the octahedral sites.

Supplementary Table 1. Structure of P3-type $Na_{2/3}Cu_{1/3}Mn_{2/3}O_2$ refined from synchrotron powder XRD data (R_{wp} =11.82%, Gof = 2.78).

S.G. $P2_1/c$, a=6.6023(2) Å, b= 8.7152(2) Å, c=5.0085(1) Å, β = 122.346(1)°						
Atom	Wyck.	*x*	*y*	*z*	Occ.	B_{eq} (Å^2)
Cu1	2c	0	0	0.5	1	0.99(5)
Mn2	4e	0.0231(2)	0.3347(2)	0.5257(3)	1	0.10(3)
Na1	4e	0.4901(5)	0.3726(4)	0.2085(6)	1	0.50(6)
O1	4e	0.1737(10)	-0.0140(7)	0.2712(12)	1	0.30(14)
O2	4e	0.2129(9)	0.3079(5)	0.3454(11)	1	0.30(11)
O3	4e	0.1658(10)	0.6827(6)	0.2815(12)	1	1.19(16)

Supplementary Table 2. Structure of P3-type $Na_{2/3}Cu_{1/3}Mn_{2/3}O_2$ refined from POWGEN neutron diffraction data. (R_{wp} =4.10%, Gof = 2.75).

S.G. $P2_1/c$, a=6.6059(9) Å, b= 8.7104(12) Å, c=5.0100(6) Å, β = 122.382(9)°						
Atom	Wyck.	*x*	*y*	*z*	Occ.	B_{eq} (Å^2)
Cu1	2c	0	0	0.5	0.854(10)	0.59(11)
Mn1	2c	0	0	0.5	0.146(10)	0.59(11)
Mn2	4e	0.0385(15)	0.3400(8)	0.5467(17)	0.927(5)	0.44(7)
Cu2	4e	0.0385(15)	0.3400(8)	0.5467(17)	0.073(5)	0.44(7)
Na1	4e	0.5003(12)	0.3679(9)	0.2226(17)	1	0.55(10)
O1	4e	0.1729(14)	-0.0149(8)	0.2722(15)	1	1.18(9)
O2	4e	0.2113(8)	0.3087(5)	0.3423(10)	1	0.24(5)
O3	4e	0.1665(9)	0.6808(6)	0.2837(11)	1	0.35(6)

Supplementary Table 3. Structure of P3-type $Na_{2/3}Cu_{1/3}Mn_{2/3}O_2$ refined from NOMAD neutron diffraction data (R_{wp} =3.92%, Gof = 6.91).

S.G. $P2_1/c$, a=6.6065(5) Å, b= 8.7032(8) Å, c=5.0103(5) Å, β = 122.381(4)°						
Atom	Wyck.	*x*	*y*	*z*	Occ.	B_{eq} (Å^2)
Cu1	2c	0	0	0.5	0.903(5)	0.60(5)
Mn1	2c	0	0	0.5	0.097(5)	0.60(5)
Mn2	4e	0.0403(7)	0.3392(5)	0.5434(9)	0.951(2)	0.10(5)
Cu2	4e	0.0403(7)	0.3392(5)	0.5434(9)	0.049(2)	0.10(5)
Na1	4e	0.4985(7)	0.3691(5)	0.2156(9)	1	0.81(8)
O1	4e	0.1804(6)	-0.0146(4)	0.2812(7)	1	1.20(5)
O2	4e	0.2105(4)	0.3071(3)	0.3442(5)	1	0.33(4)
O3	4e	0.1662(5)	0.6802(3)	0.2816(6)	1	0.47(4)

Supplementary Table 4. Selected bond lengths from the refined structure of P3-type $Na_{2/3}Cu_{1/3}Mn_{2/3}O_2$ (NOMAD data).

Bonds	Distance (Å)	Bonds	Distance (Å)
Na1-O1(×1)	2.342(7)	Cu1-O1 (×2)	2.009(5)
Na1-O1(×1)	2.421(5)	Cu1-O2 (×2)	2.271(2)
Na1-O2(×1)	2.376(6)	Cu1-O3 (×2)	1.999(2)
Na1-O2(×1)	2.376(4)	Mn2-O1(×1)	1.962(5)
Na1-O3(×1)	2.336(4)	Mn2-O1(×1)	1.851(5)
Na1-O3(×1)	2.753(6)	Mn2-O2(×1)	1.829(4)
		Mn2-O2(×1)	1.881(7)
		Mn2-O3(×1)	1.984(7)
		Mn2-O3(×1)	2.012(4)

Supplementary Table 5. Short-range structure of P3-type $Na_{2/3}Cu_{1/3}Mn_{2/3}O_2$ refined from neutron PDF data (1- 10 Å).

S.G. $P2_1/c$, a=6.6453(44) Å, b= 8.6999(49) Å, c=5.0311(28) Å, β = 122.576(45)°						
Atom	Wyck.	*x*	*y*	*z*	Occ.	B_{eq} (Å^2)
Cu1	2c	0	0	0.5	1	0.56(5)
Mn1	4e	0.0255(9)	0.3403(4)	0.5301(9)	1	0.32(5)
Na1	4e	0.5036(7)	0.3738(9)	0.2072(20)	1	1.44(15)
O1	4e	0.1759(6)	-0.0098(4)	0.2724(8)	1	1.08(5)
O2	4e	0.2151(5)	0.3143(4)	0.3499(10)	1	0.77(6)
O3	4e	0.1653(5)	0.6731(3)	0.2805(7)	1	0.90(9)

Supplementary Table 6. Structure of 3.66 V (versus Na^+/Na) charged P3-type $Na_xCu_{1/3}Mn_{2/3}O_2$ (P3' phase) refined from *ex situ* NOMAD neutron diffraction data (R_{wp} = 3.28%, Gof = 4.32).

S.G. $P2_1/c$, a = 6.6934(7) Å, b= 8.7052(8) Å, c= 5.0204(5) Å, β = 122.243(32)°						
Atom	Wyck.	*x*	*y*	*z*	Occ.	B_{eq} (Å^2)
Cu1	2c	0	0	0.5	0.903*	0.36(13)
Mn1	2c	0	0	0.5	0.097*	0.36(13)
Mn2	4e	0.0266(37)	0.3318(15)	0.5413(34)	0.951*	0.13(12)
Cu2	4e	0.0266(37)	0.3318(15)	0.5413(34)	0.049*	0.13(12)
Na1	4e	0.5523(34)	0.3341(32)	0.2624(52)	0.72(6)	2.01(78)
O1	4e	0.1548(18)	-0.0022(14)	0.3042(23)	1	0.73(12)
O2	4e	0.2086(21)	0.3028(10)	0.3495(23)	1	0.73(12)
O3	4e	0.1638(21)	0.6793(8)	0.2663(21)	1	0.73(12)

* fixed to the values in the pristine material

Supplementary Table 7. Structure of 4.6 V (versus Na^+/Na) charged P3-type $Na_xCu_{1/3}Mn_{2/3}O_2$ (O1' phase) refined from *in situ* XRD data. (R_{wp} = 5.08%, Gof = 1.13).

S.G. $P2_1/c$, a= 6.4254(5) Å, b= 8.5415(6) Å, c=5.0123(4) Å, β = 118.323(8)°						
Atom	Wyck.	*x*	*y*	*z*	Occ.	B_{eq} (Å^2)
Cu1	2c	0	0	0.5	1	0.1(3)
Mn2	4e	0.9800(12)	0.6675(8)	0.4810(44)	1	0.1(2)
Na1	2d	0.5	0	0.5	0.81(3)	0.5(2)
O1	4e	0.3013(30)	-0.0112(59)	0.0754(42)	1	0.4(3)
O2	4e	0.1773(53)	0.1627(73)	0.443(13)	1	0.4(3)
O3	4e	0.1767(50)	0.6688(73)	0.940(14)	1	0.4(3)
