# Supplementary material for: Stabilizing lattice oxygen redox in layered sodium transition metal oxide through spin singlet state
Source: Nat Commun. 2023 Nov 23;14:7665. doi: 10.1038/s41467-023-43031-6 (PMC10667238; doi:10.1038/s41467-023-43031-6)
Supplement: Supplementary file 4 — Description of Additional Supplementary Files [file 41467_2023_43031_MOESM4_ESM.pdf]

File Name: Supplementary Data 1
Description: Data and source code for the DFT calculation.

File Name: Supplementary Data 2
Description: Refined structure file (in CIF format) of P3-type $Na_{2/3}Cu_{1/3}Mn_{2/3}O_2$ using neutron Bragg diffraction data.
